# Neglected Superior Dislocation of the Shoulder Joint: A Case Report

**DOI:** 10.7759/cureus.55245

**Published:** 2024-02-29

**Authors:** Pranav Gupta, Sandeep Shrivastav, Dhananjay Gupta, Prakhar Shrivastav, Aditya Pundkar

**Affiliations:** 1 Orthopaedics, Jawaharlal Nehru Medical College, Datta Meghe Institute of Higher Education and Research, Wardha, IND; 2 Orthopaedics, Datta Meghe Institute of Higher Education and Research, Wardha, IND; 3 Orthopaedics, Fortis Flt. Lt. Rajan Dhall Hospital, New Delhi, IND

**Keywords:** glenohumeral joint dislocation, shoulder instability, shoulder trauma, irreducible shoulder dislocation, superior dislocation

## Abstract

The anterior subtype of shoulder dislocations constitutes the vast majority that either reduces instantly or is reduced at the point of care with no serious complexities. The posterior ones are infrequent and inferior and superior dislocations are even more rare. Rupture of the deltoid is considered to be linked with superior dislocation; regardless, very few articles are available pertaining to the mechanism of onset and the management of a superior shoulder dislocation. In the line of traumatic shoulder dislocations, we present a one-year-old neglected case of a 23-year-old male who sustained an open injury over the right outstretched upper arm, abducted at an angle of approximately 45° due to a fall from a height of approximately 18 feet. This unique report outlines the various surgical modalities available, given the patient's late presentation due to neglect.

## Introduction

In terms of the occurrence of all types of shoulder dislocations, a bimodal age pattern is seen, which exhibits a rise in 21 to 30 years and 61 to 80 years of age groups [1]. The shoulder joint stability is accredited to the functional relations of static (capsule, glenohumeral ligaments, glenoid labrum, glenoid concavity) and dynamic (deltoid, tendons of the rotator cuff, periscapular musculature) stabilizers. The core cause of shoulder instability is the imbalance between these stabilizers [2]. Clinically, the patient may present with his arm adducted to the chest wall, with restricted and painful movements of the affected joint when superiorly dislocated [3].

In superior dislocation of the shoulder joint, an extensive rotator cuff injury might lead to the weakening of the dynamic stabilizers, hindering the resting balance and concavity compression of the glenohumeral joint, causing the humeral head to migrate upwards due to the pulling action of the deltoid muscle [3]. Although the capsuloligamentous limit to superior-inferior translation of the glenohumeral joint is at its weakest in the 45° abduction position, the arm is generally adducted when superior dislocation occurs [4].

Our patient reported a history of fall from height one year ago, sustaining an injury over his right upper limb. After clinical and radiological examination, a diagnosis of superior dislocation of the right shoulder joint was made, which is the rarest of all the subtypes of shoulder dislocations. This case report highlights the management of the rare entity and the challenges faced due to the long-neglected history of the patient.

## Case presentation

A 23-year-old male reported to the orthopedics outpatient department with chief complaints of swelling over the right shoulder region and restriction of movement of the right shoulder joint for one year. The patient gave a history of falling from a height of approximately 18 feet with an outstretched and abducted hand where he was immediately taken to the casualty by his relatives and stabilized. The injury was neglected for almost a year. He then decided to get consulted in regard to the immobility of the affected joint. The patient was admitted for further evaluation and management.

**Figure 1:**
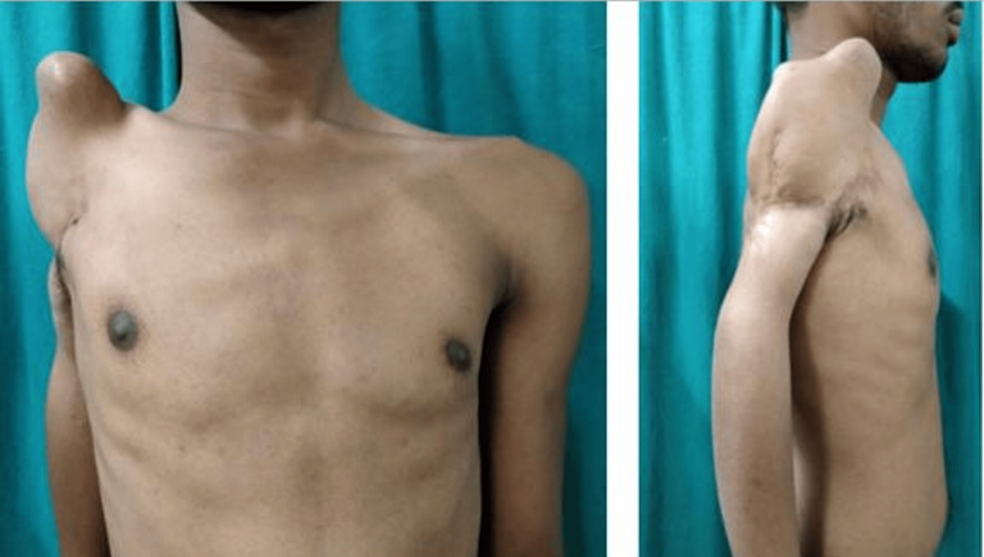
Clinical presentation of superior dislocation of the shoulder joint. Bony hard swelling over the anterosuperior part of the right upper limb extending above the level of the clavicle and anterior to the midline of the body.

After obtaining due consent, his general and systemic examinations were conducted, which were within the normal limits. He gave no history of any comorbidities. On local examination, a swelling of bony hard consistency approximately 4 × 4 cm in size was noticed. It was present over the anterosuperior part of the right arm, extending above the level of the clavicle and anterior to the midline of the body (Figure 1).

The range of movements of the right shoulder joint includes total loss of abduction, exaggerated extension of up to 70°, and no forward flexion. Contractures were present on the posterior axillary fold. Overlying soft tissue injury was also noticed. There was also wasting of the right deltoid muscle and loss of sensation in the upper lateral aspect of the arm (also known as regimental badge area anesthesia), signifying axillary nerve injury.

**Figure 2:**
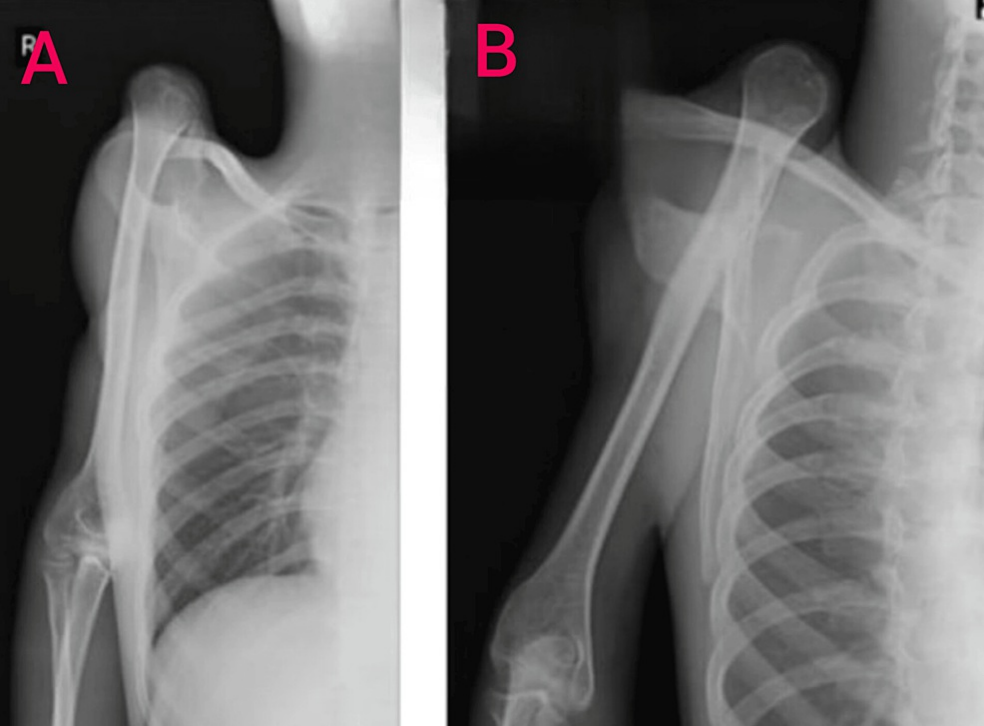
Anteroposterior (A) and lateral (B) views of the right shoulder joint. Dislocation of the head of the humerus superior to the level of the clavicle.

Radiological investigations of the right shoulder joint in anteroposterior and lateral views showed dislocation of the humeral head extending superior to the level of the clavicle. No injuries to any other structure were visible (Figure 2).

On the basis of clinical examination and radiological findings, the team came down with the provisional diagnosis of superior dislocation of the right shoulder joint.

**Figure 3:**
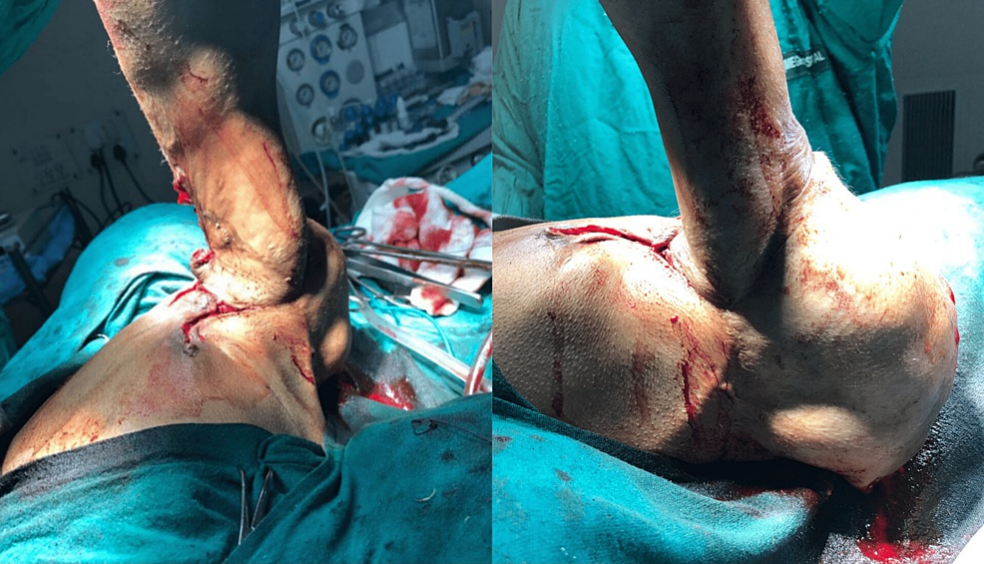
Assessment of mobility following the procedure.

After obtaining informed consent and anesthetic clearance, the patient was taken into the operation theater. The treatment was planned in two stages. In the first stage, excision arthroplasty of the right humeral head was done. The mobility of the shoulder joint was assessed after the procedure, which showed significant improvement (Figure 3).

**Figure 4:**
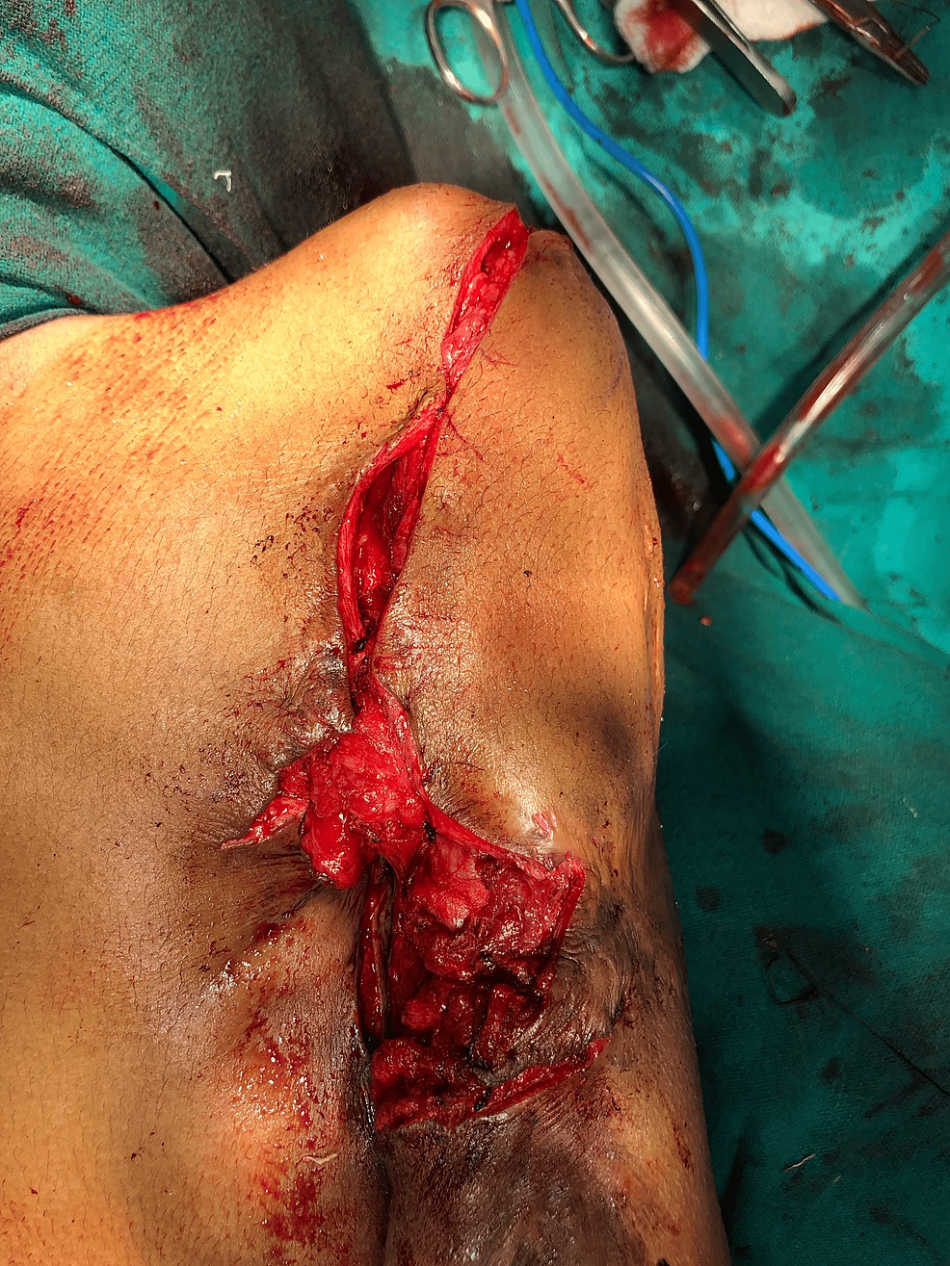
Postoperative image after release of posterior contractures.

Stage two consisted of total shoulder arthroplasty, which was not performed as the patient refused any further surgery after achieving a desirable outcome in the first procedure. Intraoperatively, tears and atrophy of the muscles of the rotator cuff (subscapularis, supraspinatus, infraspinatus, and teres minor) were also noticed. Due to the neglect, reconstruction of the damaged muscles was not possible. Finally, the contractures were also released (Figure 4).

**Figure 5:**
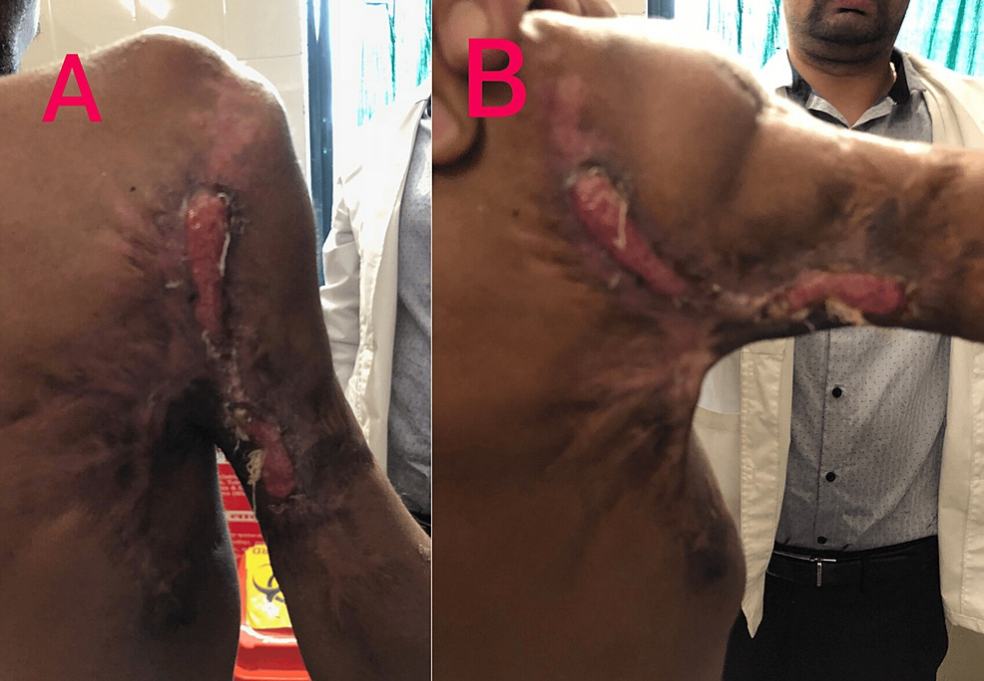
Follow-up three months after operation. (A) Active abduction of 15°, (B) further reaching 70°.

On follow-up after three months of surgery, the patient presented with regained mobility of the right shoulder joint as follows: active shoulder abduction of 15° further attained to 70°, forward flexion of 20°, an extension of 20°, and internal and external rotation of 15° and 30°, respectively (Figure 5). The patient had no fresh complaints.

## Discussion

The most common form of traumatic unidirectional glenohumeral dislocations is categorized as anterior (96.3%), followed by posterior (3%), inferior (0.5%), and superior subtype, which is considered to be extremely rare [5]. Prior articles on superior shoulder dislocation to date constitute anterosuperior dislocation, in which the head of the humerus dislocates toward the anterior acromion [4,6-8]; superolateral shoulder dislocation, in which the head of the humerus dislocates in the direction along the lateral acromion [9,10]; and locked superior shoulder dislocation, in which the dislocated head of the humerus gets locked in the area between the superior boundary of the glenoid and acromion [3].

Associated complications include fractures of the acromion, humeral tuberosities, coracoid, clavicle, or acromioclavicular dislocations, in adjunct to the neurovascular complications that we may find in shoulder dislocations [3].

The nerve most frequently and seriously injured is the axillary nerve. While the majority of axillary nerve injuries linked with glenohumeral dislocation heal without intervention, there remains a group of individuals who have a higher-grade nerve injury, who will fail to achieve an adequate recovery with ongoing functional deficit. Seddon and Sunderland's classification of nerve injuries provides insight into the pathophysiology and pathoanatomy of the injury, as well as its potential for healing. Neurapraxic injuries often have a fairly good prognosis, with full recovery occurring in 12 weeks. A higher-grade injury is indicated by axonal disruption and consequent Wallerian degeneration. The intermediate grades possess some semblance of neural architecture and therefore have an increased caliber to heal, usually within four months. The high-grade injuries have no capability to heal on their own and hence require surgical intervention, which includes surgical neurolysis, nerve grafting, and nerve transfers [11].

In the presented case report, the patient suffered from superior dislocation of the right shoulder joint, which had been neglected for a year. The manner of trauma included falling from a height onto the outstretched arm at an angle of approximately 45° of abduction. Ideally, early closed reduction should be attempted. If it fails or the shoulder instability continues, then reverse shoulder arthroplasty would have been the treatment of choice. However, due to the late presentation of the patient, complete reconstruction of the joint with full mobility was not possible. Moreover, the associated soft tissue and axillary nerve injuries were beyond repair. As a result, the team pinned down the treatment plan in two stages. The first stage involved the excision arthroplasty of the right humeral head, followed by total shoulder arthroplasty in stage two. After the first surgery, the patient regained active shoulder abduction of 15°, reaching a maximum of 70°, forward flexion of 20°, extension of 20°, and internal and external rotation of 15° and 30°, respectively. The patient refused any further surgery at this point and was satisfied with the outcome. Hence, total shoulder arthroplasty was not done.

## Conclusions

The presented case establishes important information on superior dislocation of the shoulder joint, which is the rarest of all glenohumeral dislocations, very little described in the literature. The case was further complicated by the late presentation. This report highlights the importance of early intervention to restore the complete functioning of the affected joint with the repair of the associated muscles and nerves as superior dislocation is largely predicated on rotator cuff injuries. Nerve injuries associated with shoulder dislocation need first to be recognized and consequently understood. This allows for the categorization of the injury and formulation of a plan of management. Failure of recovery of high-grade nerve injury necessitates surgical exploration. Due to the late presentation of our case, excision arthroplasty followed by total shoulder arthroplasty was planned, which was not done as per the patient’s convenience. To a certain extent, the mobility was restored; however, the muscular and axillary nerve damage could not be attended to.
